# Identification of a novel *RPS26* nonsense mutation in a Chinese Diamond-Blackfan Anemia patient

**DOI:** 10.1186/s12881-019-0848-1

**Published:** 2019-07-05

**Authors:** Xiaodong Shi, Xiaolan Huang, Yu Zhang, Xiaodai Cui

**Affiliations:** 1 Department of Haematology, Capital Institute of Paediatrics, Beijing, China; 2 Department of Key Laboratory, Capital Institute of Paediatrics, Beijing, China

**Keywords:** Diamond-Blackfan, DBA10, RPS26

## Abstract

**Background:**

Diamond-Blackfan anemia (DBA), a congenital pure red cell aplasia (PRCA), is characterized by normochromic macrocytic anemia, reticulocytopenia, and nearly absent erythroid progenitors in the bone marrow. DBA10, a subset of DBA, is an autosomal dominant disease caused by a mutation in *RPS26*. So far, there are 30 disease-causing variants in *RPS26* being reported, however, only three of them are small insert mutations.

**Case presentation:**

Here we report a three-month Chinese boy who presents with anemia from postnatal day 2. He was suspected to have Diamond-Blackfan anemia, according to the clinical result. Thus, whole-exome sequencing was performed for precise diagnosis.

**Conclusion:**

Here, a novel insert mutation c.96dupG in *RPS26* was identified by whole-exome sequencing, which caused neonatal DBA in a Chinese boy. This is the first case report of a Chinese DBA10 patient who carries a small insertion in the *RPS26* gene. These findings expand the mutation diversity of *RPS26* and demonstrate the clinical presentations of the Chinese DBA10 patient.

**Electronic supplementary material:**

The online version of this article (10.1186/s12881-019-0848-1) contains supplementary material, which is available to authorized users.

## Background

Diamond-Blackfan anemia (OMIM:613309) is an inherited pure red cell aplasia (PRCA). It is characterized by normochromic macrocytic anemia, reticulocytopenia, and a near absence of erythroid progenitors in the bone marrow. Most Diamond-Blackfan anemia (DBA) patients have growth retardation. A total of 30 to 50% of the patients present malformation in craniofacial features, upper limbs, hearts and urinary systems. Symptoms of this disease are heterogenous; they can vary even between affected family members carrying the same mutations [1]. A few genes have been reported to be related to DBA, including *RPS19*, *RPS24*, *RPS17*, *RPL35A* and *RPS26* (MIM 603701). RPS26 encodes a protein that belongs to a protein family called the ribosomal protein family. RPS26 is a crucial component of the 40S ribosome-binding site of mRNA [2], a mutation in the *RPS26* gene could significantly affect the production of the small subunit of ribosome and cause disease [3].

To date, 30 disease-causing variants in *RPS26* have been reported; only three of them are small insertion mutations. Here, we report this experience in the diagnosis of a three-month Chinese DBA patient, according to clinical and genetic test results. This boy presented anemia without any malformations from the postnatal day 2 and had no family history. A novel mutation in the *RPS26* gene was identified. To the best of our knowledge, this is the first reported Chinese DBA patient due to a small insertion in the *RPS26* gene. Our findings expand the variant diversity of *RPS26* and provide more clinical information of Chinese DBA10 patients.

## Case presentation

### Clinical information

The study was approved by the ethics committee of the Capital Institute of Paediatrics. Written informed consent was obtained from the patient’s parents for the publication of this report and any accompanying images.

The patient was a Chinese boy aged 3 months, who was admitted to the hospital because of anemia. He was born to a non-consanguineous family by normal spontaneous vaginal delivery at full-term with a birth weight of 3.03 kg. He was the only child of the family and no family history of anemia was recorded. This boy presented normochromic anemia with reticulocytopenia and normal platelet count from the postnatal day 2. The patient had no short stature or congenital malformation, and he also presented neither hepatosplenomegal nor lymphadenopathy, had no recurrent infection or bleeding during his stay in the hospital. Further laboratory examinations were performed. The blood routine was: hemoglobin 5.2 g/dL (normal range13.1–17.20 g/dL), the red blood cell (RBC) count 1.62 × 10^12^/L (reference range 4.7–6.1 × 10^12^/L) and the reticulocyte 0.43% (reference 2–6%); the mean corpuscular volume (MCV), white blood cell and platelet were in the normal range. Bone marrow examination showed a marked erythroid hypoplasia with a paucity of erythroid precursors whose ratio was 1.5%. The granulocyte system and the megakaryocyte system were normal. Testing for the antibody of blood serum, the cytomegalovirus IgM, EB virus and parvovirus B19-DNA tests were negative. No abnormalities was found according to the hemolysis checking. No thymoma was observed regarding to The X-ray and CT scan. The patient had no history of medication or exposure to harmful substances before. According to the clinical information above, he was initially diagnosed with DBA.

### Gene sequencing and data analysis

Gene sequencing was performed by Running Gene Inc. according to the manufacture’s protocol. The causal gene was discovered by proband DNA sequencing. DNA was isolated from patient’s peripheral blood using DNA Isolation Kit (Bioteke, AU1802). Concentrations of DNA samples were examined by Qubit dsDNA HS Assay Kit (Invitrogen, Q32851) on Qubit fluorometer (Invitrogen, Q33216). Then, genomic DNA were fragmented into 200-300 bp-length pieces by Covaris Acoustic System (Covaris, Massachusetts, USA). Qualified DNA fragments were then processed by following steps to build a DNA library: end-repair, A-tailing and adaptor ligation. After that, DNA libraries were prepared by pre-capture PCR. Probe-captured DNA fragments were then amplified by post-capture PCR. The final products were sequenced on Illumina HiSeq X platform (Illumina, San Diego, USA) as 150 bp paired-end reads, in accordance with its instruction manual.

Data from HiSeq X were aligned against the human reference genome (hg19) by the BWA Aligner (http://bio-bwa.sourceforge.net/). The single-nucleotide polymorphisms (SNPs) were examined using GATK software (Genome Analysis ToolKit) (https://software.broadinstitute.org/gatk/). Candidate variants were annotated using ANNOVAR (annovar.openbioinformatics.org/en/latest/). The potential impacts of single-nucleotide variants (SNVs) were predicted by MutationTaster [4], SIFT [5–7] and Polyphen-2 [8] programs. According to the ACMG guideline, all mutations were classified to be benign, likely benign, variants of unknown clinical significance (VUS), likely pathogenic and pathogenic.

The candidate causal variants identified via WES were then validated by Sanger sequencing [9]. Co-segregation analyses among the patient’s family were also performed. Amplified fragments covering mutated sites were processed using Zymoclean PCR Purification Kit (Zymo Research, USA.) and further sequenced using ABI 3730 DNA Sequencer (SeqGen, CA). Sanger sequencing data were analyzed by Chromas Lite v2.01 (Technelysium Pty Ltd., Tewantin, QLD, Australia).

After gene sequencing and data analysis, a heterozygous variant in *RPS26* gene was identified (Additional file 1: Figure S1). This mutation is c.96dupG in chr12:56436301. This frame-shift mutation would cause p.K32fsX5, producing a truncated protein with only 37 amino acids. Sanger sequencing confirmed this mutation as a de novo mutation and demonstrated that neither of the patient’s parents is carrier of the mutation. This null variant was interpreted as “pathogenic” according to the variant guidelines of ACMG based upon the following criteria: it is predicted as null variants due to frame-shift (PVS1), this mutation is a de novo variant in a family without disease history (PS2), this mutant is absent in control (PM2). Therefore, we summarized that this heterozygous null variant in *RPS26* is likely contributed to the DBA10 in this patient.

## Discussion and conclusions

Diamond-Blackfan anemia (DBA) is a congenital pure red cell aplasia. Symptoms usually appear within the first year after birth. Normochromic and macrocytic anemia, reticulocytopenia and absent erythroid progenitors in bone marrow are the main characteristics of DBA and are also used as the standards of diagnosis in DBA. Patients with DBA typically present growth retardation and multisystem malformations in 30–50% of cases [1]. Different subtypes of DBA were reported to be related to various genes. Subtypes of DBA and related genes were listed in Table 1. In several independent studies, RPS26 is responsible for 2.6–6.4% of DBA patients [3, 10, 11]. Thus, screening the causal gene is essential to achieve precise diagnosis. It also provides a possibility in gene therapy in the future.

**Table 1:** Subtypes of DBA and related genes

DBA subtypes	Gene/Locus
DBA1	RPS19
DBA2	8p23-p22
DBA3	RPS24
DBA4	RPS17
DBA5	RPL35A
DBA6	RPL5
DBA7	RPL11
DBA8	RPS7
DBA9	RPS10
DBA10	RPS26
DBA11	RPL26
DBA12	RPL15
DBA13	RPS29
DBA14	TSR2
DBA15	RPS28
DBA16	RPL27
DBA17	RPS27

RPS26 is a ribosomal protein with 115 amino acids. It is an indispensable component of the 40S subunit, which is located close to the mRNA exit site region [12]. RPS26 was demonstrated to regulate the transactivation activity of tumor suppressor p53, which is a transcription factor for genes involved in cell cycle arrest, apoptosis and cellular senescence under stress environments [13]. RPS26 was also reported to be associated with NMD regulator Upf1 in yeast, which indicates a regulatory role of RPS26 in Nonsense-mediated mRNA decay (NMD) process [2]. In addition, RPS26 was documented to be involved in mRNA-specific translation by recognition of the Kozak sequence [13]. The biogenesis of ribosomes might also be affected by RPS26, since a study shows that RPS26 was implicated in the formation of a nucleophosmin [12]. The translation of apoptosis-related protein, biogenesis of ribosomes and mRNA-specific translation may all be implicated in the pathology of DBA10.

**Fig. 1:**
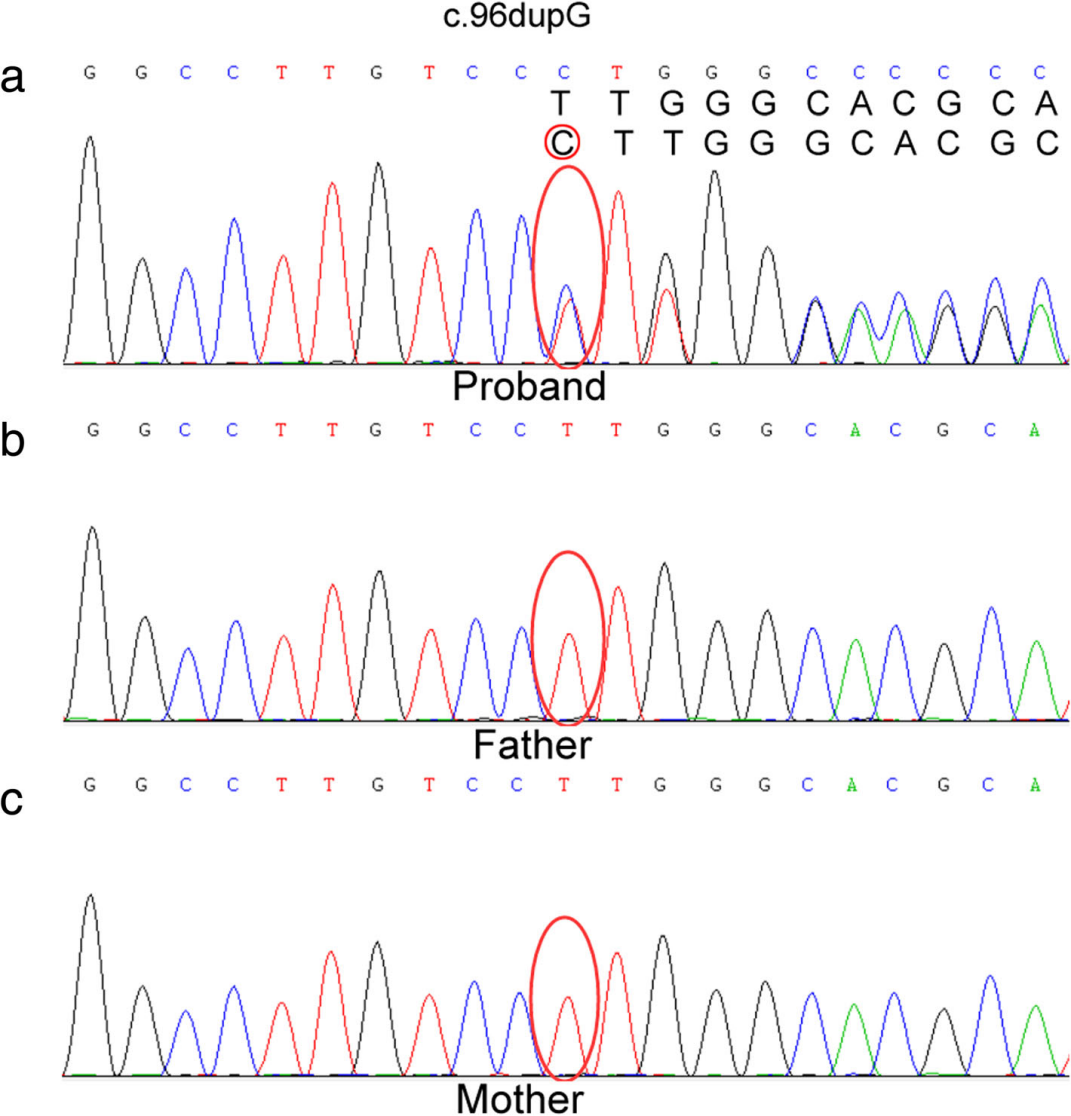
Sanger traces for PCR products of the patient and his parents. **a** Sanger traces for PCR products of the patient indicated a heterozygous insert mutation in *RPS26* (c.96dupG). Cytosine is the complementary base of Guanine. **b**, **c** Sanger traces for PCR of his parents (**b** for the proband’s father, **c** for the proband’s mother), neither of his parents carry this mutation

The mutation identified in this study is c.96dupG in the *RPS26* gene. This one nucleotide insertion results in a frame-shift mutation p.K32fsX5, producing a protein with only 37 amino acids. This truncated protein is significantly shorter than the wild-type RPS26 protein, which consists of 115 amino acids. DBA10 caused by the *RPS26* mutation is an autosomal dominant disease, which means if only one allele carries a disease-causing mutation, this individual would have the disease. Sanger sequencing revealed that neither of the child’s parents is a carrier of this mutation (Fig. 1). The mutation detected in the patient is a de novo variant. This also corresponds with the inheritance pattern of the disease.

After searching mutations of *RPS26* in HGMD database, 30 variants were found to be recorded. There are 11 point-mutations, 4 splicing mutations, 5 small deletions, 3 small insertions and 7 gross deletions. Then, all the available clinical information of DBA10 patients was summarized. In all DBA10 patients, the onset age varies from 2 weeks to 2 years and 5 months. Most patients presented symptoms of DBA within the first 2 months. Seventeen of 35 patients (48.57%) presented malformations in the skeleton, heart or urinary system. The patients are not location limited; Czech, Russian, Chinese and Japanese patients were all identified with variants in this gene [3, 14–20]. The clinical presentation of mutations is also heterogenous. For a prevalently observed mutation c.1A > G (p.M1V), in clinical records of eight patients carrying this mutation, six patients did not present any malformation [3, 16, 20]. One patient was recorded as presenting duplicated pelvocalicon on the right kidney, while another patient was recorded having winged scapula [3], vitamin D deficiency and moderate iron overload [16]. In this report, our patient carried a novel nonsense mutation c.96dupG (p.K32fsX5) and presented severe anemia only 2 days after birth, and was diagnosed as DBA by clinical and genetic diagnosis at 3.5 months. Until then, no significant malformation was observed.

In summary, we identified a novel nonsense mutation in *RPS26*, c.96dupG (p.K32fsX5) that is associated with autosomal dominant Diamond-Blackfan anemia in a two-day-old neonate. This mutation can result in the production of a truncated protein with 37 amino acids. However, the precise effect of this mutation on protein structure and function remains unknown. Further investigation of mutation effect and the pathology of gene *RPS26* on DBA still needs to be carried out.

## Additional file

Additional file 1: **Figure S1.** Next-Generation Sequencing result of the proband. The BAM file of the NGS sequencing data presented a heterozygous insert mutation of c.96dupG in *RPS26* gene. (TIF 7219 kb)

## Data Availability

We did not use new software, databases, or applications/tools in the manuscript, all results and figures have already provided in the manuscript.

## Abbreviations

ACMG: American College of Medical Genetics and Genomics
DBA: Diamond-Blackfan anemia
MCV: Mean corpuscular volume
NMD: Nonsense-mediated mRNA decay
PRCA: Pure red cell aplasia
