# Supplementary figures and images for: Identification of a novel RPS26 nonsense mutation in a Chinese Diamond-Blackfan Anemia patient
Source: BMC Med Genet. 2019 Jul 5;20:120. doi: 10.1186/s12881-019-0848-1 (PMC6612111; doi:10.1186/s12881-019-0848-1)

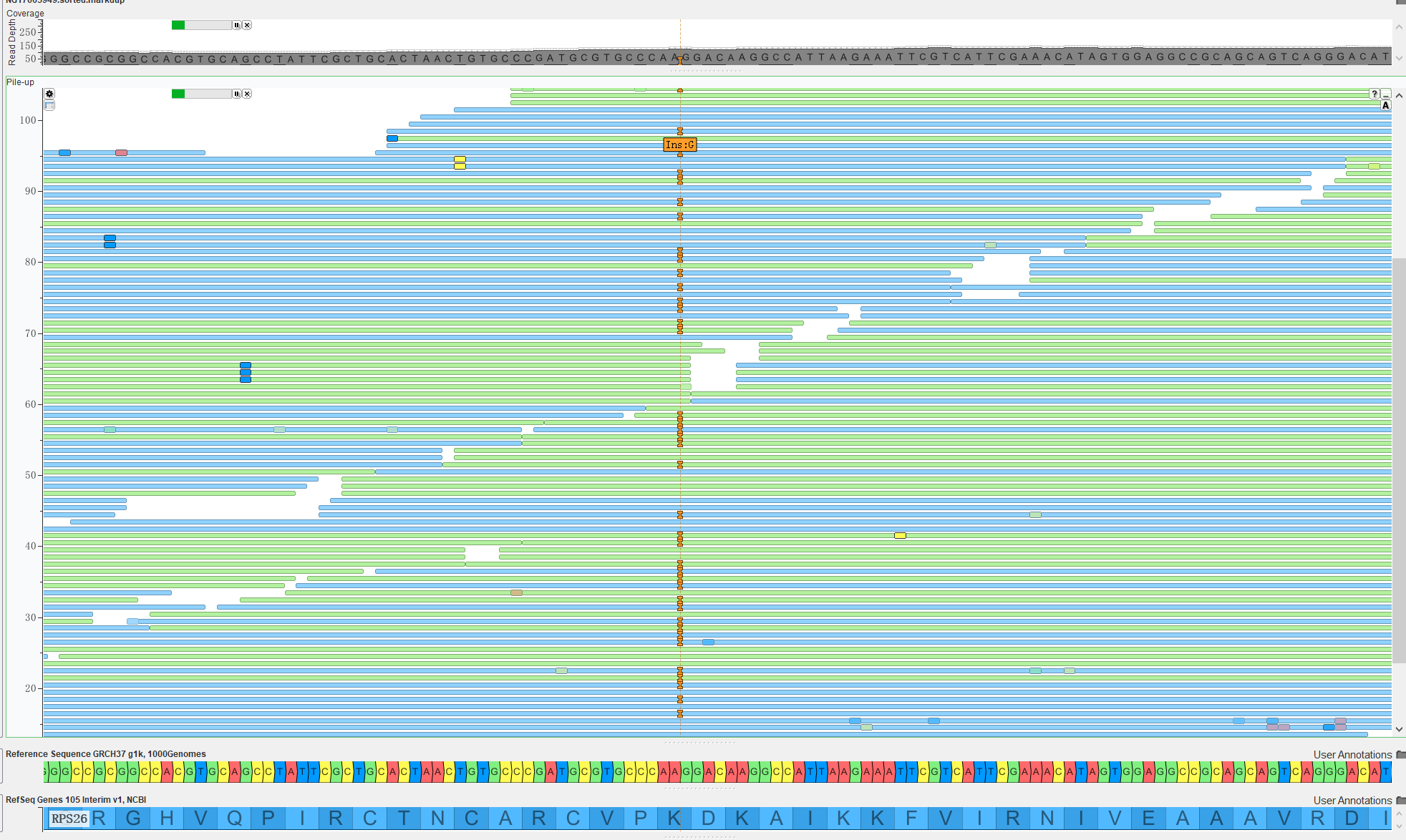

Supplement: Supplementary file 1 — Figure S1. Next-Generation Sequencing result of the proband. The BAM file of the NGS sequencing data presented a heterozygous insert mutation of c.96dupG in RPS26 gene. (TIF 7219 kb) [file 12881_2019_848_MOESM1_ESM.tif]
